# Decline in Morel Production upon Continuous Cropping Is Related to Changes in Soil Mycobiome

**DOI:** 10.3390/jof9040492

**Published:** 2023-04-20

**Authors:** Yan Zhang, Sifan Sun, Didi Luo, Ping Mao, Rusly Rosazlina, Francis Martin, Lingling Xu

**Affiliations:** 1 School of Biological Sciences, Universiti Sains Malaysia, Minden 11800, Penang, Malaysia; 2 Key Laboratory of Plant-Microbe Collaboration, School of Biological and Environmental Engineering, Xi’an University, Xi’an 710065, China; 3 Université de Lorraine, INRAE, Interactions Arbres/Microorganismes, F-54000 Nancy, France

**Keywords:** *Morchella sextelata*, mycobiome, continuous cropping, fungal community, soil physicochemical properties

## Abstract

The black morel *Morchella sextelata* (Morchellaceae, Pezizales) is a valuable edible mushroom that can be cultivated on a large scale, but a severe yield decline is observed during continuous cropping. The effect of long-term cropping on soil-borne diseases and the dysbiosis of the microbiome and how these biotic factors affect the morel yield are not well understood. To help fill this knowledge gap, we designed an indoor experiment to investigate the influence of black morel cropping regimes on soil physicochemical properties, richness and distribution of fungal community, and morel primordial production. In this study, we employed rDNA metabarcoding and microbial network analysis to evaluate the effect of non-continuous and continuous cropping regimes on the fungal community during three developmental stages of black morel production, namely, the bare soil mycelium, mushroom conidial, and primordial stages. The results showed that during the first year, *M. sextelata* mycelium overwhelmed the resident soil fungal community by reducing the alpha diversity and niche breadth of soil fungal patterns by a greater amount compared to the continuous cropping regime, leading to high crop yield of 12.39 ± 6.09/quadrat but less complex soil mycobiome. To sustain continuous cropping, exogenous nutrition bags and morel mycelial spawn were consecutively added to the soil. The additional nutrient input stimulated the growth of fungal saprotrophic decomposers. The degrading activity of soil saprotrophs, including *M.* *sextelata*, caused a significant increase in soil nutrient content. This led to an inhibitory effect on the formation of morel primordia, resulting in a sharp decline to 0.29 ± 0.25/quadrat and 0.17 ± 0.24/quadrat, respectively, in the final morel cropping. Our findings provided a dynamic overview of the soil fungal community during morel mushroom production, allowing us to identify beneficial and detrimental fungal taxa in the soil mycobiome involved in morel cultivation. The information acquired from this study can be applied to mitigate the adverse impact of continuous cropping on the yield of black morel.

## 1. Introduction

Black morels (*Morchella* spp., Morchellaceae, Pezizales) are widely distributed in temperate regions of China [1,2,3]. These edible ascomycetous mushrooms are of high economic and scientific value [2,3]. In recent years, several black morel species have been “domesticated” and used in large-scale production of highly prized mushroom crops [4]. The black morel agroindustry is now expanding rapidly, both in China and around the world [3,5,6], but a severe decline in morel yields and resistance to soil-borne diseases have been observed as a result of continuous cropping, hampering the full development of the morel agroindustry [1,7].

Long-term continuous cropping affects soil microbial diversity and community composition, thereby leading to negative impacts on soil productivity and health [1,7]. Unlike most edible fungi, grown in pre-sterilized substrate packages, *M. sextelata* is cultivated directly into soil beds, hence its mycelial growth and fruiting are impacted by the interacting soil resident fungal community [1,2,8,9]. Previous studies have reported that morel yield was positively correlated with the alpha-diversity of the soil fungal community [8], and several prominent species act as soil-borne pathogens for black morels [1]. The proliferation of pathogenic fungi, such as *Penicillium*, *Trichoderma*, and *Aspergillus*, appears to be the main cause of the decline in *M. sextelata* production in the second year of continuous cropping cultivation [7]. The latter works mainly focused on the production of fruiting bodies. On the other hand, little is known about the dynamics of the fungal community during the earliest stages of the life cycle [10] of *M. sextelata*, that is, the mycelial and conidial development and primordium formation, during continuous cropping.

Characterizing simultaneous changes in soil composition and the fungal community during consecutive croppings of *M. sextelata* development can reveal the complex and dynamic relationships between soil fungal succession and the mushroom yield reduction resulting from continuous cropping. Owing to the entanglement of biotic and abiotic factors taking place in soils of large-scale morel farm production, we developed a controlled indoor cropping system for the black morel. This included non-continuous cropping bed soils sown with isolate HX13 (referred to as NCC) of *M. sextelata*, continuous cropping bed soils sown with isolate HX13 (referred to as CC), and continuous cropping bed soils sown with isolate YN05 (referred to as CCi), respectively. Then, we followed the simultaneous changes in soil physicochemical properties, fungal community composition, and structure of microbial networks at three different morel developmental stages (bare soil, conidial, and primordial stages). Our results provide novel insights on the impact of the fungal community (i.e., the mycobiome) on *M. sextelata* primordium formation and fruiting yield during continuous cropping. These data can be further used to design scenarios to alleviate the detrimental effects of soil-borne factors and fungal taxa affecting morel production yields.

## 2. Materials and Methods

### 2.1. Experimental Settings and Cultivation Protocol

**Figure 1:**
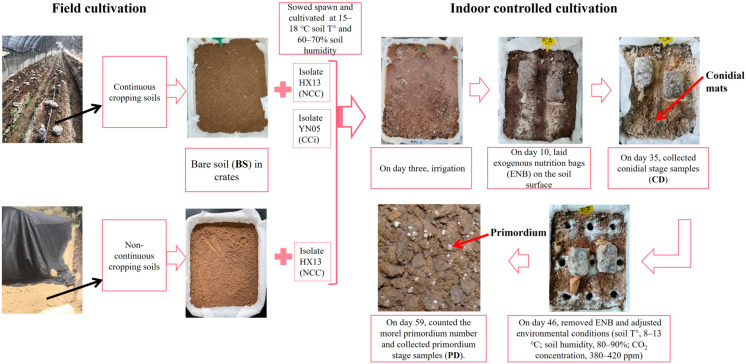
Schematic diagram of the experimental design.

In June 2019, a greenhouse structure was built on a vacant farmland located in Hu County, Shannxi Province, northwestern China (N 34°6′31.05″, E 108°36′18.04″) without applying herbicides, fungicides, pesticides, or chemical fertilizers to the soil [8]. In the subsequent two years, production of black morel (*M. sextelata*, isolate HX13) fruiting bodies was conducted following a continuous cropping regime [7]. After fruit bodies were harvested in April 2020, the top soil layer (0 to 30 cm) of the mushroom beds was collected in January 2021 at 20 random sites across the greenhouse (that is, continuous cropping soils). For control samples corresponding to non-continuous cropping soils, we collected the top 0 to 30 cm layer of bare soils outside the greenhouse (i.e., no cultivation of morels) (Figure 1). Soil samples were then transferred to the laboratory for further use in indoor cultivating studies.

After air-drying to remove the excess moisture, soil samples from the greenhouse were mixed thoroughly and then sieved through 2-mm mesh to remove plant debris and stones [1,11]. A non-woven fabric pad (50 g/m^2^) was placed on the bottom of a plastic turnover basket crate (43 cm × 33 cm × 10 cm) (n° 17, Xulang, China), and the latter was filled with 12 kg of sieved soil (Figure 1). Black morel isolates HX13 and YN05, known for their high production yield, were used as inoculum in these experiments. To evaluate the effects of cultural protocols and spawn strains on soil physicochemical properties and the microbial community, three cropping regimes were implemented: (1) bare soil sown with HX13 (that is, no previous cropping, referred to as NCC), (2) continuous cropping soil from the greenhouse sown with isolate HX13 (CC), and (3) continuous cropping soil from the greenhouse sown with isolate YN05 (CCi). Bare soil (BS) was used as the control. Four replicates were conducted per treatment.

Morel cultures were carried out as follows. Crates were filled with 12 kg of NCC or CC soils, and two trenches were dug at the middle of every crate at a depth of 3–5 cm. Then, 75 g quicklime (Quicklime powder, Chuanhui, China) was added together with 250 g of morel spawn [5]. The inoculum was covered with soil, and 75 g of wood ash (Lu Wan Tian, Fumin, China) were added. The cultures were conducted at 15–18 °C soil temperature and 60–70% humidity. Three days after sowing, the crates were irrigated slowly for at least 12 h until the soil was completely saturated with water. On day 10, two exogenous nutrition bags (ENBs, containing 330 g substrate [5]) were laid on the soil surface and covered by mulch to maintain the temperature, humidity, and dim sunlight. On day 35, white and powdery conidial mats [12] emerged from the soil surface, and soil samples were collected (referred to as conidial stage samples (CD)). On day 46, the ENBs were removed, and drop watering was applied for at least 12 h to trigger morel fructification. Environmental conditions were then adjusted (soil T°, 8–13 °C; soil humidity, 80–90%; CO2 concentration, 380–420 ppm) to promote morel primordium production. On day 59, tiny and semitransparent morel primordia were counted, and soil samples were collected (referred to as primordium stage samples (PD)). The number of morel primordia per quadrat (6 cm × 6 cm) was used to estimate the primordium yield.

### 2.2. Soil Sampling

A total of 36 soil samples were collected (3 cropping regimes (NCC, CC, CCi) × 4 replicates × 3 stages (BS, CD, PD)). Soil cores of the BS, CD, and PD stages were harvested by using a plastic soil sampler (2.5 cm in diameter). A total of nine soil cylinder cores were taken from each crate at every stage (Figure 1), and quicklime was sprinkled in the holes caused by soil digging to prevent potential microbial contamination at stages CD and PD (Figure 1). The soil in the crate at stage BS was remixed after the soil samples were collected. The soil cores collected from every crate were mixed thoroughly, and 15 g of mixed soil was snap frozen in liquid N_2_ and stored at −80 °C for further metabarcoding analysis [8]. The remaining soil was dried to assay the soil physicochemical properties.

### 2.3. Soil Physicochemical Analysis

Analyses for soil pH, organic matter (OM) content, total nitrogen (TN), alkali-hydro nitrogen (AN), total phosphorus (TP), available phosphorus (AP), and total potassium (TK) were performed according to NY/T 1377-2007, NYT 1121.4-2006, TN 53-1987, alkaline hydrolysis-diffusion method [13], NY/T 88-1988, NY/T 1121.7-2014, and NY/T 87-1988, respectively.

### 2.4. DNA Extraction, PCR Amplification, and Sequencing

The soil DNA was extracted from the soil samples using the TIANamp Soil DNA Kit (TIANGEN, Beijing, China). The primers ITS1-F (5′-CTTGGTCATTTAGAGGAAGTAA-3′) and ITS2-R (5′-GCTGCGTTCTTCATCGATGC-3′) [14,15] were used to amplify the rDNA internal transcribed spacer (ITS) region of fungi. PCR products were purified with the Qiagen Gel Extraction Kit (QIAGEN, Hilden, Germany). The paired-end sequencing of the amplicons was performed on the Illumina NovaSeq sequencer of Novogene Biotechnology Co., Ltd. (Tianjing, China).

Sequencing data were processed using the QIIME pipeline software [16]. Fungal sequences were trimmed and assigned to different samples based on their barcodes. The sequences were then clustered into OTUs based on 97% identity [17]. The representative sequence alignments were generated using MUSCLE [18]. To process the taxonomic classification of OTUs, the representative sequences of each OTU were generated and aligned against the RDP databases [19] and UNITE database [20], respectively. The relative abundance data for taxa were generated based on the read count for each taxon across samples by using the total-sum scaling method. All raw sequence data were deposited in the National Center for Biotechnology Information (NCBI) database with BioProject accession number PRJNA935967.

### 2.5. Statistical Analysis

Statistical analyses were conducted in R v4.2.2 [21] and SPSS version 23.0 software (SPSS Inc., Chicago, IL, USA). To comprehensively evaluate the alpha-diversity within the community, we analyzed several indices at the OTU level. These included the Chao 1 index, which estimates the number of organisms [22]; Faith’s phylogenetic index, which measures the taxonomic richness diversity [23]; and Pielou’s evenness index, which quantifies the evenness of the community [24]. The beta diversity analyses of the fungal composition were determined by visual assessment using principal coordinate analysis (PCoA) plots [25] and permutational multivariate analysis of variance (PERMANOVA) [26]. In order to evaluate the resources available to the NCC, CC, and CCi microbial communities, we calculated the community-level niche breadth (that is, habitat specialization) using the spaa package [27]. The distribution of generalist, specialist, and neutral taxa was calculated by the EcolUtils R package [28]. Pearson’s correlation analyses were employed to correlate the morel primordium yield with soil physicochemical properties. OTUs that were confirmed by indicator species analysis with the R package indicspecies [29] were defined as cropping-sensitive OTUs (csOTUs). The sum relative abundance of OTUs was larger than 0.01. The greedy optimization of modularity algorithm was utilized as implemented in igraph [30]. The descriptive and topological network properties were calculated with igraph. Keystone OTUs were identified as those OTUs with a degree larger than 70 and an eigenvector centrality larger than 0.9 for each network. To examine the relationships between modules and environmental factors, we calculated the module eigengene E (the first principal component of modules) of the top module for the NCC, CC, and CCi networks and then tested their relationships with physicochemical properties using Spearman’s rank correlation test [31]. One-way analysis of variance was performed using SPSS 23.0 software to test physicochemical properties, alpha diversity, and niche breadth at a 0.05 significance level (SPSS Inc., Chicago, IL, USA).

## 3. Results

### 3.1. Relationships between Primordium Yield and Soil Physicochemical Characteristics

**Figure 2:**
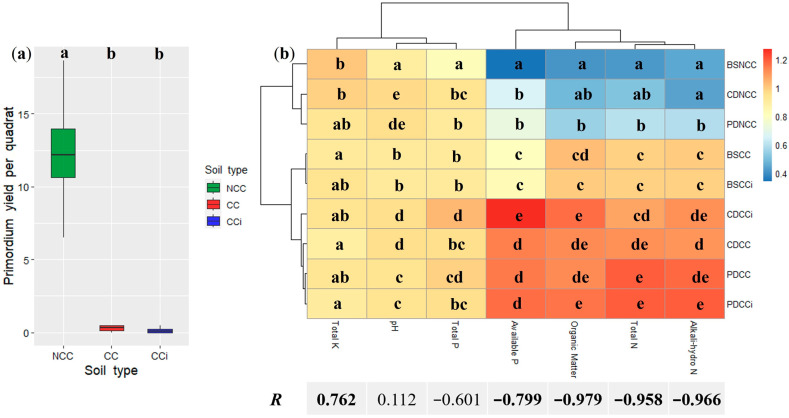
Primordium yield and physicochemical characteristics under different cropping regimes. (**a**) Primordium yield. All values are indicated as the mean ± SE. Bars with different letters represent significant differences among different treatments (*p* < 0.05); (**b**) Physicochemical characteristics and their Pearson’s correlation with primordium yield. Data are mean values of *n* = 4; Lower case letters denote significant differences among different treatments at *p* < 0.05. Significant correlation coefficients are noted in bold font, where *p* < 0.05.

The *M. sextelata* primordium yields were 12.39 ± 6.09/quadrat, 0.29 ± 0.25/quadrat, and 0.17 ± 0.24/quadrat for NCC, CC, and CCi, respectively, confirming that continuous cropping resulted in a striking decline in morel production for both spawn isolates (Figure 2a). Major changes in soil physicochemical characteristics were observed during the time course of mycelial growth and fruiting (Figure S1). Pearson’s correlation analysis revealed that organic matter content, total N, alkali-hydro N, and available P were negatively related to morel primordium yields (*p* < 0.05), whereas total K was positively correlated (*p* < 0.05; Figure 2b). The mushroom cultivation led to an increased content of organic matter, total N, alkali-hydro N, and available P, while total K decreased (Figure S1). With the exception of available P, which was significantly higher in CCi than in CC at stage CD (Figure 2b), no difference in physicochemical properties was found between CC and CCi, suggesting that the genotype of the spawn isolate had no major impact on soil physicochemical characteristics.

### 3.2. Changes in Fungal Richness and Community Composition

**Figure 3:**
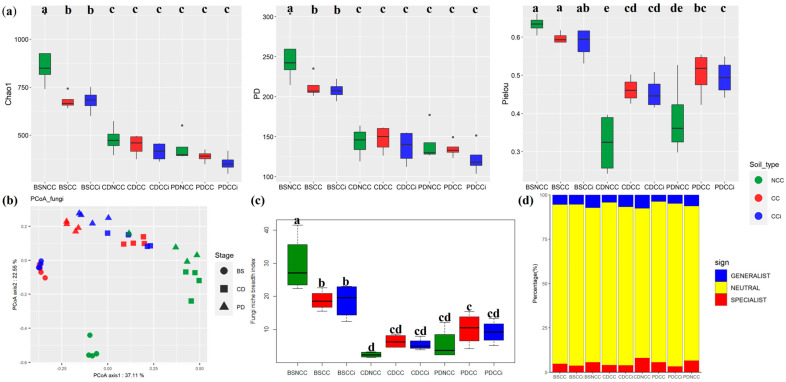
Temporal dynamics of diversity and distribution patterns of crop-associated microbiomes in NCC, CC, and CCi systems at BS, CD, and PD stages. (**a**) The diversity of the microbial community associated with *M. sextelata* as indicated by Chao1, Faith’s phylogenetic diversity, and Pielou’s evenness index. All values are indicated as the mean ± SE. The different lowercase letters indicate significant differences (*p* < 0.05, Duncan); (**b**) PCoA analysis of soil microbial community associated with *M. sextelata* based on the Bray distance metric. The percentage value for each axis represents the proportion of total variation explained. Samples collected from NCC, CC, and CCi are shown in green, red, and blue, respectively. Circles, squares, and triangles separately represent bare soil (BS), conidial stage (CD), and primordial stage (PD), respectively; (**c**) Boxplots showing mean niche breadth in soil fungal communities. Lower letters represent *p* < 0.05; (**d**) Relative contributions of habitat generalists and specialists in soil fungal communities.

We followed the changes in the soil fungal community during *M. sextelata* fruiting in the three cropping regimes. Sequencing of the rDNA ITS yielded 2,352,645 sequences, ranging between 60,411 and 69,829 sequences per sample (median 65,811; Table S1). In total, we identified 2255 fungal OTUs across all soil samples. The alpha diversity of the soil fungal communities decreased along the three morel developmental stages in all cropping systems (Figure 3a). The fungal diversity was higher in bare (BS) and NCC soils, and significantly decreased after inoculating the morel spawn (stages CD and PD) in all cropping regimes (NCC, CC, and CCi; Figure 3a). No significant difference was found between CC and CCi treatments. As shown by PCoA and PERMANOVA (Bray-Curtis dissimilarities), the variations in fungal community were mainly explained by the morel developmental stage (R^2^ = 33.31%, *p* < 0.001) and cropping regime (R^2^ = 19.31%, *p* < 0.001), then by isolate type (R^2^ = 2.49%, *p* = 0.25; Figure 3b and Table 1).

Initially, the niche breadth of the soil fungal community was highest in the bare soil (BS) in NCC, followed by the BS in CC and CCi (Figure 3c). After the morel spawn were inoculated, the niche breadth markedly decreased in the three cropping regimes (Figure 3c). No significant difference was found between CC and CCi. The proportion of both generalists and specialists in CC and CCi decreased compared to NCC soils across all stages (Figure 3d).

### 3.3. Cropping-Sensitive OTU Identification, Co-Occurrence Networks, and Key Taxa

**Figure 4:**
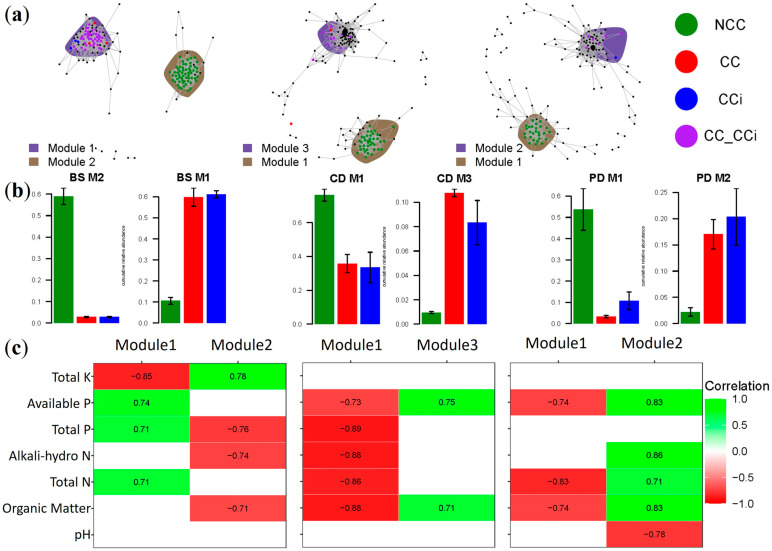
Co-occurrence patterns of cropping-sensitive OTUs. (**a**) Co-occurrence networks visualizing significant correlations (ρ > 0.7, *p* < 0.05; indicated with gray lines) between OTUs in soil. OTUs are colored by their association with the different cropping systems (black OTUs are insensitive to cropping practices). Shaded areas represent the network modules containing csOTUs; (**b**) Cumulative relative abundance of the cropping sensitive modules in NCC, CC, and CCi soil networks (*y*-axis reported cumulative relative abundance). NCC (forest green), CC (red), CCi (blue); (**c**) Spearman’s correlations between modules and environmental variables in NCC, CC, and CCi. Only significant correlations (*p* < 0.05) are shown.

To explore the extent to which cropping regimes impacted the co-occurrence patterns in the fungal community across morel developmental stages, separate co-occurrence networks for the three stages were constructed, and their properties were determined for OTUs with significant correlations (ρ > 0.7, *p* < 0.05; Figure 4 and Table 2). Indicator species analysis was employed to identify individual fungal OTU in soil communities whose abundance varied significantly among different treatments and which were defined as cropping-sensitive OTUs (csOTUs). Two discrete modules containing high proportions of csOTUs were identified in microbial networks across the three stages (Figure 4a). Interestingly, the module composition was specific to cropping regimes. For example, the effect of NCC was apparent with a discrete module, such as module #2 in stage BS and module #1 in stages CD and PD, containing csOTUs specific to the NCC system and defined as an NCC responsive module (Figure 4a,b). Intriguingly, most CC- or CCi-specific csOTUs were shared (CC-Cci) at every stage, which was reminiscent of the effects seen in the previous PcoA analyses (Figure 3b). Similar to the NCC-responsive modules, the CC-responsive modules containing csOTUs specific to CC or Cci regimes (including CC, Cci, or CC-CCi) were all located in the other modules at every stage, such as module #1 in stage BS, module #3 in stage CD, and module #2 in stage PD (Figure 4a). No shared OTUs were found between NCC and CC or NCC and CCi (Figure 4a).

**Figure 5:**
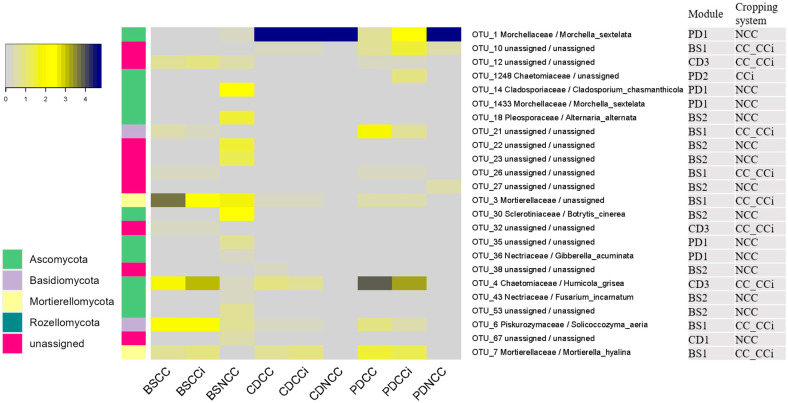
Mean relative abundances of cropping-sensitive OTUs identified by indicator species analysis. OTUs are labeled with their family/species level taxonomy assignment, with the phylum level taxonomy assignment indicated by the colored bars.

The csOTUs with the higher relative abundance (within the top 50 most abundant fungal OTUs) in NCC-responsive modules were *M. sextelata* (OTU1, OTU1433), *Cladosporium chasmanthicola* (OTU14), *Alternaria alternata* (OTU18), *Botrytis cinerea* (OTU30), *Gibberella acuminata* (OTU36), and *Fusarium incarnatum* (OTU43; Figure 5). The csOTUs with the higher relative abundance in CC-responsive modules were *Humicola grisea* (OTU4), *Mortierellaceae* (OTU3), *Solicoccozyma aeria* (OTU6), *Mortierella hyalina* (OTU7), and *Trichocladium* (OTU1248; Figure 5). Note that *Trichocladium* is a phragmoconidial counterpart of *Humicola* [32].

Compared to NCC-responsive modules, CC-responsive modules comprised more OTUs but fewer csOTUs at every stage (Table 2), suggesting a key role for csOTUs in the morel production. Furthermore, the OTUs, average degree, and connectivity network wide of the CC-responsive modules showed that the complexity and stability of the co-occurrence networks decreased markedly after the inoculation of morel spawn at stages CD and PD (Table 2), suggesting that a reduction in microbial network complexity is accompanying the morel fructification.

We further investigated the correlations between modules and physicochemical properties with Spearman’s correlation analysis (Figure 4c). Surprisingly, all of the NCC-responsive modules were negatively correlated with organic matter content, total N, alkali-hydro N, and available P (Figure 4c). Meanwhile, module #2 in stage BS was positively related to total K content. Conversely, all of the CC-responsive modules were positively correlated with organic matter content, total N, alkali-hydro N, and available P, and module #1 in stage BS was negatively related to TK (Figure 4c). These results were also consistent with the previous correlation analysis between physiochemical properties and morel primordium yield (Figure 2b), implying that the changes in soil mycobiome were related to the fluctuation of soil physicochemical properties and morel development.

Several key OTUs were recognized as important drivers of microbial community structure and function. They are csOTUs, also sensitive to cropping regimes (Table 2). More keystone OTUs were identified in stage BS than in stages CD and PD in both NCC- and CC-responsive modules (Table S2). Keystone taxa found in NCC-responsive modules comprised OTU173 (*Septoriella phragmitis*), OTU36 (*Gibberella acuminata*), OTU96 (Helotiaceae), and OTU94 (Ascomycota) in stage BS; OTU100 (Mortierellaceae), OTU579 (Ascomycota), and OTU59 (Phaeosphaeriaceae) in stage CD; and OTU1378 (*Mortierella*) in stage PD. On the other hand, the keystone OTUs identified in CC-responsive modules were OTU47 (Herpotrichiellaceae) and OTU78 (*Dichotomopilus*) in stage PD (Table S2). Only OTU36 (*Gibberella acuminata*) was an abundant keystone OTU also listed in the top 50 most abundant OTUs, and the remaining keystone taxa were all rare OTUs (Table S2).

## 4. Discussion

The continuous cropping of black morels (*M. sextelata*) currently used in agro-industrial facilities is leading to a drastic decline in mushroom yield [5]. Previous studies aimed to identify the factors involved in the reduction of morel fructification by collecting samples from large-scale continuous cropping cultivation soils [1,8] and characterizing the soil microbial communities during the fruiting body production [7]. However, conflicting results were obtained between studies, for example, whether the soil microbial diversity is the major reason for the difference between successful fructification and lack of fructification; what the exact role of chemical variables in morel fruiting is [7,8]; and at what stage of the development of morels does the outbreak of pathogenic fungi occur. Therefore, further experiments are still needed to examine the impact of continuous cropping on the soil mycobiome and soil physicochemical properties at different cultivation stages. Owing to the fact that morel cultivation is carried out in farmlands, with fluctuating biotic and abiotic factors (e.g., climate, soil types, cultivation regimes, material handling by farmers), we developed controlled experimental settings where only the cropping scheme and morel spawn isolates varied. This simpler experimental design allowed us to unveil the changes taking place in the distribution of soil fungal species during morel production in both NCC and CC systems at different *M. sextelata* developmental stages. These changes in the mycobiome composition were also correlated to alterations in soil physicochemical properties.

The present study revealed that continuous cropping of black morels significantly altered the soil physicochemical characteristics of the mushroom beds with an increased content in organic matter, total N, alkali-hydro N, and available P, while total K decreased. The addition of the morel spawn and ENBs, containing large amounts of wheat seeds and corn cobs, likely explained these striking changes in soil nutrient levels. It is known that the saprotrophic *M. sextelata* secretes a diverse set of degrading enzymes involved in the substrate decomposition [4]. This leads to a rapid increase in the organic carbon content in the surface soil of the mushroom bed, which is thereafter catabolized during mycelial growth to sustain fruiting body yield [33]. Many morel growers measure N and P contents in soil before spawn inoculation to avoid using beds with high contents of nutrients [34]. High soil fertility is beneficial to the morel mycelial growth and conidial production before fructification [33,34], but it appears to be detrimental to the differentiation of morel primordia. These results also explain why ENBs are used in artificial cultivation of black morels instead of adding all of the ingredients of the ENBs into the soil directly. The addition of ENBs to the mushroom bed resolved the dilemma between the high nutrition requirement by proliferating mycelium and conidial formation and low nutrient level, triggering fructification, whereas the high nutrient accumulation in soil resulting from continuous cropping of black morels made the soil not suitable for fructification, reducing yields and leading to crop failure.

The black morel species abundantly fruit on post-fire habitats during spring or summer and are thus termed “burn morels” [35]. In China, to simulate the natural post-fire environment, wood ash, a kind of natural potash fertilizer with a high content of water-soluble K, is often used as preferred fertilizer for large-scale black morel cultivation. In our experiments, we also added wood ash after sowing the black morel spawn, either in this indoor experiment or in the previous two-year outdoor continuous cultivation. Nevertheless, the total K content steadily decreased during black morel cultivation over time (Figure S1f), suggesting that a large amount of K supplementation is required during the growth and development of black morels. In conclusion, bed soils with low organic matter, N, and P content but high K (e.g., natural potash fertilizer) will likely support a high production of morel primordia.

The inoculation of bed soils by *M. sextelata* mycelial spawn resulted in a lower alpha diversity of soil fungal taxa. The decrease was more pronounced in NCC by comparison to CC and CCi. The genetic background of the *M. sextelata* isolate had no significant effect on fungal community composition. As shown by the “niche breadth” analysis estimating the habitat specialization of taxa [36], OTUs occurred in fewer habitats after morel spawn sowing. The decline of the niche breadth of soil fungal taxa was correlated with the decrease in taxonomic richness (Chao1 and PD indices) and evenness (Pielou’s evenness index). After sowing, *M. sextelata* rapidly dominated the bed soil mycobiome, eliminating the other fungal members. This competitive capability was stronger in NCC than in CC or CCi, suggesting that a stronger suppression of the soil resident fungi was needed to guarantee a high primordium production. The decline in alpha diversity and niche breadth resulted in an imbalance of the soil mycobiome, which may explain the observed alterations in soil physicochemical properties and sharp decline in morel primordium yield in the continuous cropping cultivation scheme.

Cropping-sensitive fungi, csOTUs, were identified in NCC, CC, and CCi across every stage. They are indicator taxa explaining the various β-diversity patterns observed in the different cropping regimes. For example, most csOTUs in CC and CCi systems were shared, consistent with the result that no significant difference was found between the treatments inoculated with different isolates (Table 1). Surprisingly, except for *M. sextelata*, all of the abundant csOTUs in NCC-responsive modules are known as plant pathogenic fungi (Figure 5), including *Fusarium incarnatum*, inducing severe stipe rot disease on *M. importuna* [37]. These pathogenic fungi were abundant only in bare soil (BS). After *M. sextelata* sowing, the proportion of these pathogenic OTUs in inoculated soils markedly decreased, suggesting that the morel mycelium invaded NCC soils, depressed the resident pathogenic fungi, and occupied the soil niche as the dominant invader.

For CC-responsive modules, abundant csOTUs were plentiful in CC and CCi regimes across all three stages, except for OTU1248 (*Trichocladium*), which was only abundant at the PD stage in the continuous cropping regime. These findings suggest that the presence of these taxa may partly explain the detrimental effects of continuous cropping. The CC-responsive Mortierellaceae species are soil-dwelling saprobes widely known to assist crops in P acquisition and contribute to pools of long-term stable organic matter [38,39]. *Mortierella* species are known dominant inhabitants of fructification-suppressed soils of black morels [8]. Organic amendments change the fungal community composition, with significant increase in relative abundance of *M. elongata* in the bulk and rhizosphere soils [39]. *M. hyalina* acts as an N cycling saprotroph in many environments [40]. *Solicoccozyma aeria* is involved in biodegradation in agricultural soils [41]. *Humicola grisea* and *Trichocladium* are known as cellulolytic fungi [42], and they may be favored by the high cellulose content of the ENBs and spawn substrates [4]. Importantly, *M. sextelata* itself is a strong decomposer of substrates found in ENBs and spawn substrates, resulting in a rapid increase in the organic carbon content in inoculated soils.

Potential morel fungal pathogens have been detected in high proportions in bed soils with low or no morel yields and in production based on continuous cropping [7,8]. In our survey, no known morel pathogens were identified among the abundant csOTUs in CC or CCi. This could be related to the fact that previous studies were conducted at the fruiting body production stage, while our experiment focused on the primordium stage, suggesting that the pathogen load may increase at the morel fruiting stage. In addition, previous studies were conducted in farmlands or greenhouses, full of various microbes, including morel pathogens. Finally, many potential morel pathogenic fungi, such as *Penicillium*, *Trichoderma*, *Fusarium,* and *Aspergillus* [7], are also widespread and common opportunistic pathogens in the soil environment. Undoubtedly, they were detected in all of the samples in our experiment but accounting for a low proportion of the mycobiome, even in CC and CCi. However, dissemination of these fungi likely increases during the fruiting period because fruiting bodies are a rich source of nutrients; their growth takes place at a higher temperature (18–22 °C vs. 8–13 °C) for longer cultivation time (20–40 days vs. 8–15 days) than the primordium period. Furthermore, the morel abundance in CC and CCi systems was much lower in CC and CCi than in NCC, especially at stage PD (Figure 5).

In brief, although csOTUs in NCC were mainly plant or morel pathogens, they were only abundant in the BS stage and decreased significantly after the black morels were inoculated, while csOTUs in CC or CCi were generally decomposers, enhancing soil fertility, which was positive to plant growth and production but inhibited the primordium differentiation of black morels, thus leading to the CC obstacles.

## 5. Conclusions

Our findings clearly demonstrated that continuous cropping of *M. sextelata* led to an increase in organic matter, total N, alkali-hydro N, and available P and a decrease in total K in inoculated soil samples. The alpha diversity and niche breadth were significantly decreased after inoculating the morel spawn, especially in the NCC system. The beta diversity was mainly explained by the developmental stage and cropping regime. The cropping-sensitive OTUs in the continuous cropping regime were generally decomposers. Co-occurrence microbial network analysis further revealed that the fungal module composition was specific to the cropping regime. Additionally, compared to the NCC regime, the complexity of co-occurrence networks was significantly reduced in CC regimes. It was the increase of soil nutrition, as well as the dysbiosis of soil microbiome, rather than the accumulation of fungal pathogens, that caused the continuous cropping obstacle of *M. sextelata*. Our study provides a framework for predicting the alterations in the microbial community and the resulting changes in the beneficial and detrimental interactions between the morel mycelium and the rest of the fungal community during a continuous cropping regime.

## Figures and Tables

**Table 1:** Results of PERMANOVA testing of the effects of *Cropping regime* (NCC, CC, and CCi), *Stage* (BS, CD, and PD), and *Isolate* (HX13 and YN05) on fungal communities. Significant effects are indicated in bold (*** *p* < 0.001).

	Fungi Pseudo-F	P	R^2^ (%)
Isolate	1.2744	0.2535	2.49
Cropping regime	19.5925 ***	0.0001	19.31
Stage	16.9015 ***	0.0001	33.31
Isolate·Stage	1.2264	0.2598	4.80
Cropping regime·Stage	5.181 ***	0.0001	10.21

**Table 2:** Properties of fungal co-occurrence networks.

Community	Modules	^1^ OTUs	^2^ Connections	^3^ Connectivity Network Wide	Average Degree	^4^ Keystone (csOTU)	^5^ csOTU (%)	csOTU Affiliation
BS	Module2	67	842	12.57	25	9 (9)	59 (88.1)	NCC
	Module1	81	1098	13.56	27	6 (6)	45 (55.6)	CC, CCi, CC-CCi
CD	Module1	58	450	7.76	16	4 (4)	39 (67.2)	NCC
	Module3	63	922	14.63	30	0 (0)	8 (12.7)	^6^ CC-CCi
PD	Module1	54	268	4.96	10	1 (1)	27 (50)	NCC
	Module2	65	606	9.32	19	3 (3)	7 (10.8)	CC, CCi, CC-CCi

^1^ Number of nodes in target module; ^2^ Number of edges in target module; ^3^ Mean number of connections per node; ^4^ Number of keystone OTUs (number of keystone taxa belonging to csOTU); ^5^ Number of csOTUs present in the target module (percentage of csOTUs in OTUs); ^6^ The csOTUs shared by both CC and Cci.

## Data Availability

All raw sequence data have been deposited to NCBI with BioProject accession number PRJNA935967.

## Supplementary Materials

The following supporting information can be downloaded at https://www.mdpi.com/article/10.3390/jof9040492/s1: Figure S1: Line charts of physicochemical characteristics significantly related to morel primordial yield during black morel cultivation; Table S1: The number of high-quality sequences in every replicate; Table S2: Keystone OTUs identified in soil fungal communities documented with taxonomy assignments, stage, modules, OTU IDs, degree of co-occurrence values, eigenvector centrality, group, csOTU status, and top50.
