# Supplementary material for: Decline in Morel Production upon Continuous Cropping Is Related to Changes in Soil Mycobiome
Source: J Fungi (Basel). 2023 Apr 20;9(4):492. doi: 10.3390/jof9040492 (PMC10143708; doi:10.3390/jof9040492)
Supplement: Supplementary file 1 [file jof-09-00492-s001.zip › jof-2281683-supplementary.pdf]

Supporting Information

Supplementary figures

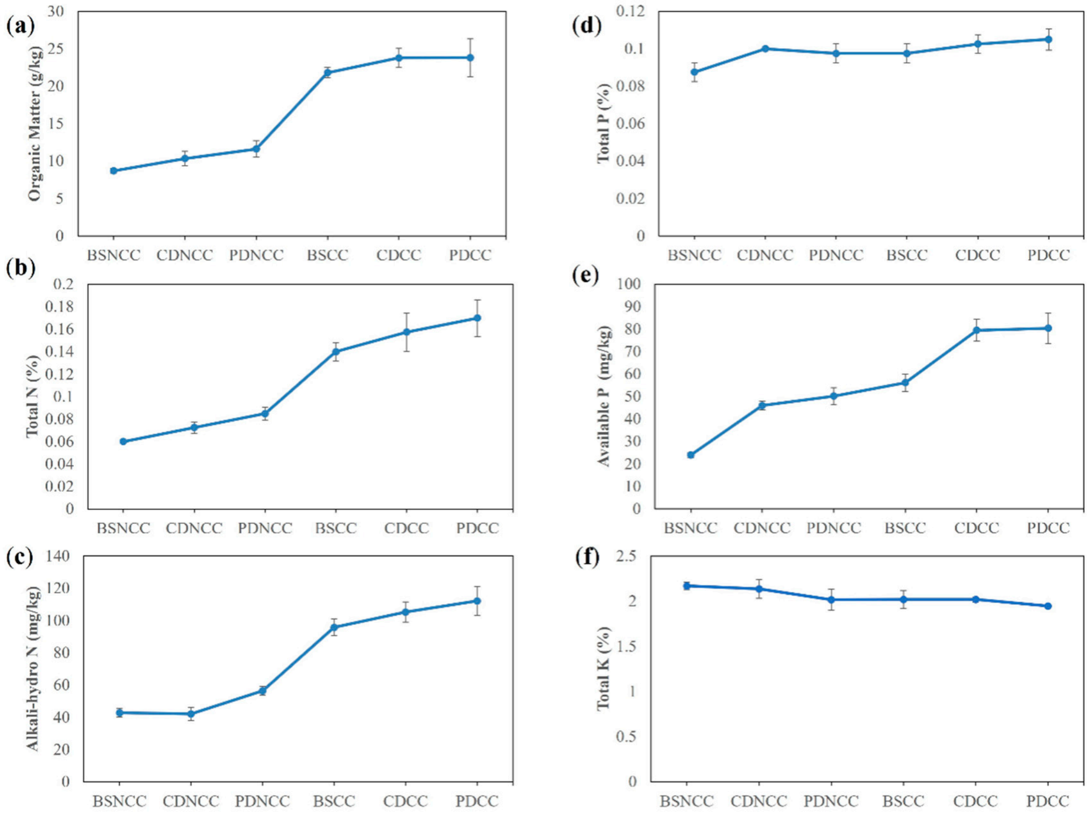

Figure S1. Line charts of physicochemical characteristics significantly related to morel primordial yield during black morel cultivation. (**a**) Organic Matter; (**b**) Total N; (**c**) Alkali-hydro N; (**d**) Total P; (**e**) Available P; (**f**) Total K

Supplementary tables

Table S1. The number of high-quality sequences in every replicate.

Sample Name	Fungi high-quality sequences
BSNCCA	64,532
BSNCCB	63,617
BSNCCC	69,617
BSNCCD	66,821
BSCCA	65,567
BSCCB	62,457
BSCCC	60,797
BSCCD	69,778
BSCCiA	60,690
BSCCiB	60,411

BSCCiC	62,218
BSCCiD	69,280
CDNCCA	64,665
CDNCCB	69,829
CDNCCC	61,906
CDNCCD	66,996
CDCCA	65,636
CDCCB	66,848
CDCCC	67,164
CDCCD	66,195
CDCCiA	69,294
CDCCiB	66,655
CDCCiC	60,439
CDCCiD	65,986
PDNCCA	66,035
PDNCCB	69,384
PDNCCC	64,454
PDNCCD	65,536
PDCCA	66,816
PDCCB	62,383
PDCCC	61,792
PDCCD	69,714
PDCCiA	68,599
PDCCiB	67,020
PDCCiC	62,169
PDCCiD	61,345
**Sum**	**2352645**
**Min**	**60411**
**Max**	**69829**
**Median**	**65811**

Table S2. Keystone OTUs identified in soil fungal communities documented with taxonomy assignments, stage, Modules, OTU IDs, degree of co-occurrence values, eignevector_centrality, group, whether or not csOTU and top50.

Stage	Modules	Node name	Degree	Eignevector_centrality	Group	Phylum	Class	Order	Family	Genus	Species	csOTU	top50
BS	2	OTU_173	106	0.9076	BSNCC	Ascomycota	Dothideomycetes	Pleosporales	Phaeosphaeriaceae	Septoriella	Septoriella_phragmitis	Yes	
	2	OTU_36	95	0.9523	BSNCC	Ascomycota	Sordariomycetes	Hypocreales	Nectriaceae	Gibberella	Gibberella_acuminata	Yes	+
	2	OTU_96	84	0.9493	BSNCC	Ascomycota	Leotiomycetes	Helotiales	Helotiaceae	unassigned	unassigned	Yes	
	2	OTU_94	97	0.9047	BSNCC	Ascomycota	unassigned	unassigned	unassigned	unassigned	unassigned	Yes	
	2	OTU_122	96	1.0000	BSNCC	unassigned	unassigned	unassigned	unassigned	unassigned	unassigned	Yes	

	2	OTU_183	98	0.9181	BSNCC	unassigned	unassigned	unassigned	unassigned	unassigned	unassigned	Yes	
	2	OTU_23	89	0.9314	BSNCC	unassigned	unassigned	unassigned	unassigned	unassigned	unassigned	Yes	+
	2	OTU_231	78	0.9287	BSNCC	unassigned	unassigned	unassigned	unassigned	unassigned	unassigned	Yes	
	2	OTU_82	99	0.9294	BSNCC	unassigned	unassigned	unassigned	unassigned	unassigned	unassigned	Yes	
	1	OTU_1032	93	0.9395	BSCC_BSCCi	unassigned	unassigned	unassigned	unassigned	unassigned	unassigned	Yes	
	1	OTU_25	89	0.9243	BSCCi	unassigned	unassigned	unassigned	unassigned	unassigned	unassigned	Yes	+
	1	OTU_62	95	0.9144	BSCC_BSCCi	unassigned	unassigned	unassigned	unassigned	unassigned	unassigned	Yes	
	1	OTU_64	95	0.9057	BSCC_BSCCi	unassigned	unassigned	unassigned	unassigned	unassigned	unassigned	Yes	
	1	OTU_72	96	0.9075	BSCC_BSCCi	unassigned	unassigned	unassigned	unassigned	unassigned	unassigned	Yes	
	1	OTU_75	95	0.9503	BSCC_BSCCi	unassigned	unassigned	unassigned	unassigned	unassigned	unassigned	Yes	
CD	1	OTU_100	75	0.9999	CDNCC	Mortierellomycota	Mortierellomycetes	Mortierellales	Mortierellaceae	unassigned	unassigned	Yes	
	1	OTU_579	98	0.9001	CDNCC	Ascomycota	unassigned	unassigned	unassigned	unassigned	unassigned	Yes	
	1	OTU_59	74	0.9201	CDNCC	Ascomycota	Dothideomycetes	Pleosporales	Phaeosphaeriaceae	unassigned	unassigned	Yes	
	1	OTU_65	86	0.9425	CDNCC	unassigned	unassigned	unassigned	unassigned	unassigned	unassigned	Yes	
PD	1	OTU_1378	75	0.9501	PDNCC	Mortierellomycota	Mortierellomycetes	Mortierellales	Mortierellaceae	Mortierella	unassigned	Yes	
	2	OTU_47	82	0.9135	PDCC_PDCCi	Ascomycota	Eurotiomycetes	Chaetothyriales	Herpotrichiellaceae	unassigned	unassigned	Yes	
	2	OTU_78	80	0.9402	PDCC_PDCCi	Ascomycota	Sordariomycetes	Sordariales	Chaetomiaceae	Dichotomopilus	unassigned	Yes	
	2	OTU_121	80	0.9952	PDCC_PDCCi	unassigned	unassigned	unassigned	unassigned	unassigned	unassigned	Yes	
